# Third Harmonic Generation in Thin NbOI_2_ and TaOI_2_

**DOI:** 10.3390/nano14050412

**Published:** 2024-02-23

**Authors:** Tianhong Tang, Deng Hu, Di Lin, Liu Yang, Ziling Shen, Wenchen Yang, Haiyang Liu, Hanting Li, Xiaoyue Fan, Zhiwei Wang, Gang Wang

**Affiliations:** 1 Centre for Quantum Physics, Key Laboratory of Advanced Optoelectronic Quantum Architecture and Measurement (MOE), School of Physics, Beijing Institute of Technology, Beijing 100081, China3120221486@bit.edu.cn (D.H.);; 2 Beijing Key Lab of Nanophotonics and Ultrafine Optoelectronic Systems, Beijing Institute of Technology, Beijing 100081, China

**Keywords:** two-dimensional transition metal oxide iodides, harmonic generation, nonlinear optics, NbOI_2_, TaOI_2_

## Abstract

The niobium oxide dihalides have recently been identified as a new class of van der Waals materials exhibiting exceptionally large second-order nonlinear optical responses and robust in-plane ferroelectricity. In contrast to second-order nonlinear processes, third-order optical nonlinearities can arise irrespective of whether a crystal lattice is centrosymmetric. Here, we report third harmonic generation (THG) in two-dimensional (2D) transition metal oxide iodides, namely NbOI_2_ and TaOI_2_. We observe a comparable THG intensity from both materials. By benchmarking against THG from monolayer WS_2_, we deduce that the third-order susceptibility is approximately on the same order. THG resonances are revealed at different excitation wavelengths, likely due to enhancement by excitonic states and band edge resonances. The THG intensity increases for material thicknesses up to 30 nm, owing to weak interlayer coupling. After this threshold, it shows saturation or a decrease, due to optical interference effects. Our results establish niobium and tantalum oxide iodides as promising 2D materials for third-order nonlinear optics, with intrinsic in-plane ferroelectricity and thickness-tunable nonlinear efficiency.

## 1. Introduction

The nonlinear optical processes in 2D van der Waals materials are typically substantial, enabling the electrically controlled second harmonic generation (SHG) [1], acousto-optic-modulated pulse generation [2], and on-chip wavelength conversion [3]. The monolayer and few-layer transition metal dichalcogenides (TMDC) possess diverse optical properties, stemming from their visible and near-infrared bandgaps. The inversion symmetry breaking in monolayer TMDCs gives rise to a large second-order nonlinearity [4,5]. Additionally, the pronounced excitonic effects allow the dynamic tuning of nonlinear process at resonant energies [6,7,8,9]. Owing to their atomic-scale thickness, phase-matching conditions are usually relieved in 2D materials, permitting strong nonlinear effects [10]. These advantages have positioned 2D materials as a promising platform for integrated nonlinear photonic applications [3,11,12,13,14,15,16]. The optical harmonic generation process, especially SHG and THG, has been extensively investigated in 2D materials, including graphene, TMDCs, hexagonal boron nitride (hBN), and their stacked heterostructures [4,6,17,18,19,20].

Optical harmonic generation is one of the typical nonlinear optical (NLO) processes that arises upon the intense optical driving of materials. Thus, the induced polarization responses scale nonlinearly with the applied optical field. In an *n*-th order harmonic process, *n* photons at the fundamental frequency ω interact concurrently with the NLO medium to create one photon at the frequency *n*ω. Consequently, the efficiency of harmonic generation generally decreases for the increasing order *n*. In contrast to even-order harmonic generation (*n* = 2, 4, 6…), odd-order harmonic generation (*n* = 3, 5, 7…) could occur regardless of the centrosymmetry of the crystal lattice. Therefore, third-order processes serve as a ubiquitous probe of the intrinsic nonlinear response for most materials. The third-order nonlinear polarization $P^{\left(3\right)}\left(t\right)$ can be described as follows [21]:

(1) $$P^{\left(3\right)}\left(t\right)=\varepsilon_{0}\chi^{\left(3\right)}E^{3}\left(t\right)$$

where $\varepsilon_{0}$ is the permittivity of free space, $\chi^{\left(3\right)}$ denotes the third-order nonlinear susceptibility, and $E\left(t\right)$ is the incident electric field. As a typical third-order nonlinearity, the third harmonic generation (THG) has proven superior to commonly used PL, Raman and SHG mapping in resolving grains and boundaries in large-scale materials [22]. Moreover, ultrafast third-order nonlinear process in the femtosecond scale holds potential for telecommunication, quantum photonics and optical sensing applications. This encompasses processes like THG [19], four-wave mixing (FWM) [23], self-phase modulation (SPM) [24] and stimulated Raman scattering [25].

Transition metal oxide dihalides MOX_2_ (M = V, Nb, Ta, Mo; X = Cl, Br, I) have recently emerged as new members of 2D van der Waals materials, exhibiting unique physical properties [26,27,28,29,30,31,32]. Among them, NbOI_2_ has garnered special interest for its ferroelectricity and robust second-order optical nonlinearity. Due to the 1D Peierls distortion, the atomic displacements of Nb atoms occur along both the b and the c axis, yielding low crystallographic symmetry. However, only distortion along the b axis induces spontaneous electric polarization, while the c axis results in alternating Nb–Nb bonding [28]. In contrast, TaOI_2_ possesses first-order Peierls distortion solely along the c-axis and lacks ferroelectricity due to higher C2/m symmetry [33]. The structural similarity to NbOI_2_ suggests that TaOI_2_ may also exhibit substantial nonlinearity, though the SHG should be absent. While second-order phenomena, including SHG, sum-frequency generation (SFG), and spontaneous parametric down-conversion (SPDC) [28,30,31] in MOX_2_ have been widely studied, observations of the third-order nonlinear process are still lacking.

In this work, we report the THG in NbOI_2_ and TaOI_2_ under ambient conditions. Through comparing with the THG intensity of NbOI_2_, TaOI_2_ and WS_2_ under the same experimental parameters, we extract the effective third-order nonlinearity susceptibility, $\chi_{eff}^{\left(3\right)}$, on an order of ~$10\times 10^{-19}m^{2}/V^{2}$, which is comparable to classical 2D materials (e.g., hBN, black phosphorus, ReS_2_). THG excitation spectroscopy reveals an enhancement peak at around 1580 nm for NbOI_2_, indicating the possible resonance effects at excitonic states or the band edges. For TaOI_2_, two maximums at around 1410 nm and 1595 nm are observed. By varying the thickness of samples, we find that the THG intensity scales quadratically below 30 nm for the two materials, suggesting the weak interlayer coupling.

## 2. Materials and Methods

High-quality large NbOI_2_ and TaOI_2_ single crystals were grown via the chemical vapor transport (CVT) method, using I_2_ as a transport agent. NbOI_2_ crystals were synthesized from Nb powder (Macklin, Shanghai, China, 99.99%), Nb_2_O_5_ powder (Aladdin, Shanghai, China, 99.99%), and iodine pieces (Alfa Aesar, Haverhill, MA, USA, 99.99%). The raw materials, with a total mass of 0.5 g, were mixed in the stoichiometric ratio Nb:O:I = 1:1:2 and sealed under vacuum (~10^−3^ Pa) into quartz tubes (7 mm inner diameter, 9 mm outer diameter, 220 mm length), with all manipulations, except for the sealing procedure, being performed in an Ar-filled glove box. The sealed quartz tube was placed into a horizontal dual-temperature zone tube furnace with the raw material in the hot side. The two heating zones were heated to 600 °C in 10 h and maintained at a constant temperature for 120 h, followed by cooling down to 310 °C/240 °C over a period of 240 h in the hot/cold sides, respectively, and finally, cooled naturally to room temperature. This small temperature gradient ensures the growth of high-quality crystals of large sizes [34]. Eventually, rectangular single crystals of NbOI_2_ of large size (~4 × 8 × 2 mm^3^) were obtained in the cold zone. TaOI_2_ crystals were synthesized using a similar method to that employed for NbOI_2_, with Ta powder (Alfa Aesar, Haverhill, MA, USA, 99.98%), Ta_2_O_5_ powder (Aladdin, Shanghai, China, 99.99%), and iodine pieces (Alfa Aesar, Haverhill, MA, USA, 99.99%) as starting materials. The two-zone tube furnace was heated to 650 °C over a period of 12 h and maintained at a constant temperature for 120 h, followed by cooling down to 360 °C/290 °C, over a 240 h period for the hot/cold sides, respectively. Finally, rectangular single crystals of TaOI_2_ with a size of about 1 × 7 × 0.6 mm^3^ were obtained in the hot zone. Both NbOI_2_ and TaOI_2_ single crystals are air-stable.

XRD characterization: the powder XRD patterns were obtained using a Bruker (Billerica, MA, USA) D8 advance X-ray powder diffractometer with the Cu-Kα target at the angle of 5–80°.

Exfoliation and transfer of the thin flakes: the thin flakes were mechanically exfoliated by adhesive tape from a bulk crystal. Then, the flakes were transferred onto a 285 nm SiO_2_/Si or quartz substrate, using polydimethylsiloxane (PDMS). The transparent substrate enabled characterization without the interference effects of the substrate. The thickness of the flakes was confirmed by atomic force microscopy (Cypher S, Oxford instruments, Abingdon, UK) measurements.

Harmonic generation measurements: the experimental setup is shown in Supplementary Figure S1. The SHG and THG measurements were performed using back-reflection geometry. For THG, femtosecond pulses from a mode-locked Ti: sapphire oscillator (Chameleon Ultra II, Coherent Inc., Saxonburg, PA, USA) were focused on the sample through a 40× reflective objective (LMM40X-P01, Thorlabs, Newton, NJ, USA). For SHG, the fundamental light was from a mode-locked picosecond super continuous laser (SC-PRO, YSL photonics, Wuhan, China) and filtered via an acousto-optic tunable filter. The SHG signals were collected by a 50× objective (Nikon MUE31500, Tokyo, Japan), and both the SHG and THG signals were coupled into a multimode fiber leading to the spectrometer. The SHG and THG signals were finally dispersed in a spectrometer and detected with a silicon charge-coupled device.

## 3. Results and Discussion

**Figure 1:**
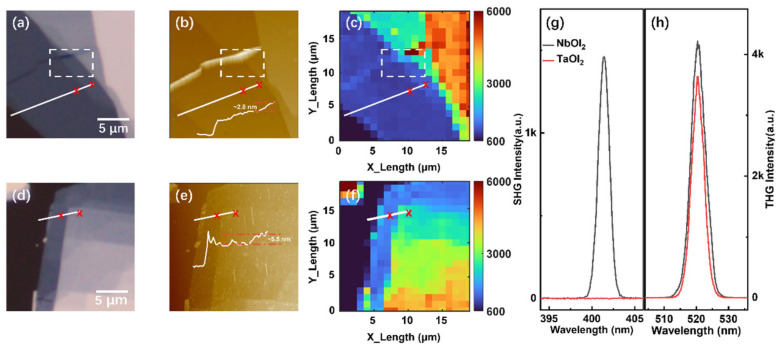
Characterization of NbOI_2_ and TaOI_2_. (**a**,**d**) Optical image of NbOI_2_ and TaOI_2_, respectively. (**b**,**e**) AFM image of NbOI_2_ and TaOI_2_, respectively. The red dashed line represents the relative height between the red x on the white line. The wrinkle in NbOI_2_ is marked by white dashed box. (**c**,**f**) THG mapping of NbOI_2_ and TaOI_2_, respectively. (**g**) Typical SHG spectra of NbOI_2_ and TaOI_2_, with the fundamental wavelength at 803 nm, respectively. (**h**) Typical THG spectra of NbOI_2_ and TaOI_2_ with the fundamental wavelength of 1560 nm, respectively.

Several NbOI_2_ and TaOI_2_ flakes of varying thicknesses were fabricated and investigated. Figure S2a,c present the powder X-ray diffraction pattern of NbOI_2_ and TaOI_2_. We confirmed peaks for NbOI_2_: at 12.2° (200), 24.4° (400), 36.9° (600), 49.9° (800), 63.6° (1000), and 78.4° (1200). Similarly, TaOI_2_ displayed peaks at 12° (200), 24.1° (400), 36.5° (600) 49.4° (800), 63° (1000), and 77.5° (1200), respectively. Both show a preferred crystal orientation along the a-axis. The sharp full width at half maximum (FWHM) of 0.05° at the (600) peak in Figure S2b,d indicates that NbOI_2_ and TaOI_2_ have good crystal quality. Figure 1a,d display the optical images of the typical samples on quartz substrates. Optical contrast is generally used to identify the thickness of thinner samples by using a silicon substrate with a specific thickness of the SiO_2_ layer [35]. However, it is hard to determine directly the thickness of the thicker samples by using optical contrast, as the differences become faint. The atomic force microscopy (AFM) measurements shown in Figure 1b,e confirm the investigated NbOI_2_ and TaOI_2_ samples, with thickness spanning from 12.7 to 44 nm across the imaged areas.

The harmonic generation was widely used to discriminate crystal orientations, thickness, and domain configurations [36,37]. As shown in Figure 1g, a comparable THG is observed from the two materials. However, the detectable SHG is demonstrated only in NbOI_2_ under identical excitation conditions, as shown in Figure 1h, which is consistent with the centrosymmetric nature of TaOI_2_ [33]. Figure 1c,f present the THG intensity mapping over the same sample zones. The thickness variation and boundary can be clearly resolved, including the ~3 nm step marked in Figure 1b (white line cut). The AFM results show a wrinkle in the white dashed box. Correspondingly, we found a five times enhancement of THG under the wrinkle, which is similar to the previous work on wrinkle-induced SHG enhancement. We attribute this phenomenon to the wrinkle-induced built-in piezoelectric field. Thus, THG can distinguish well the wrinkles in the sample. These results further highlight harmonic generation as a powerful tool for 2D materials due to its higher spatial resolution compared to linear optical techniques [38].

**Figure 2:**
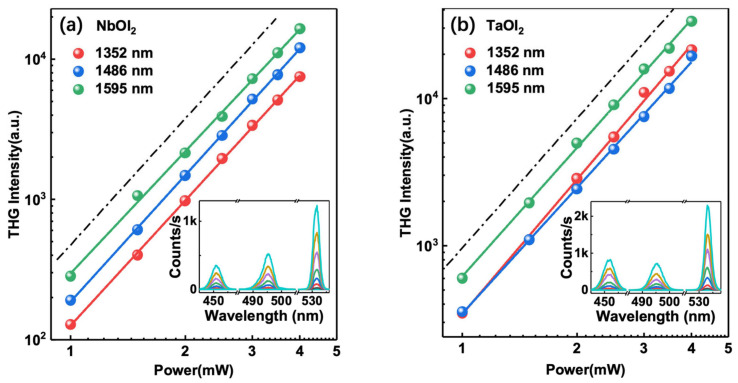
Fundamental power dependence of THG signal for (**a**) NbOI_2_ and (**b**) TaOI_2_ excitation at 1352, 1486, and 1595 nm. The black dot dashed line serves as a guide for a slope of ~3. The inset illustrates the power dependence THG spectra for NbOI_2_ and TaOI_2_ under the three excitation wavelengths. The excitation average powers changes from 1 to 4 mW corresponds to the line color of black to cyan.

The insets of Figure 2a,b present representative THG spectra of different fundamental excitation wavelengths at the telecommunications band. The THG peaks emerge at one-third of the fundamental wavelengths, confirming the third-order nonlinear optical process. Here, the sample thicknesses were 22 nm and 20 nm for NbOI_2_ and TaOI_2_, respectively. Figure 2a,b present the power dependence of the two samples on a double logarithmic scale, so that the nonlinear process can be easily identified by slope. Over this excitation power range, we carefully checked the intensities before and after the high-power experiments, and no damage was observed. The dashed guideline represents the expected cubic (slope of 3) behavior. Consistent with Equation (1), THG scales as the cube of incident power for both samples, affirming the third-order nature of the process. Upon changing of the fundamental wavelength from 1352 to 1595 nm, the THG signals increase for both NbOI_2_ and TaOI_2_, indicating the wavelength dependence of the effective $\chi^{\left(3\right)}$. 

**Figure 3:**
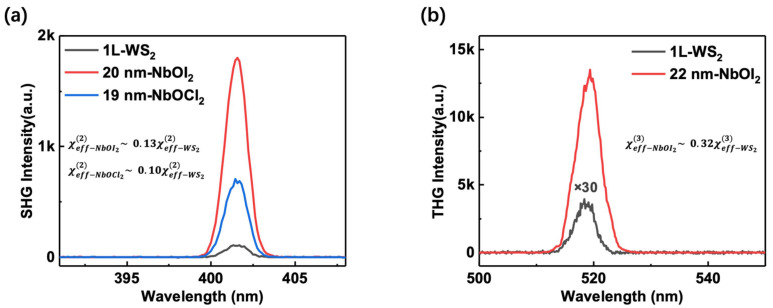
Harmonic generation spectra of different materials under the same conditions. Here, the monolayer NbOI_2_ and NbOCl_2_ are taken at 0.73 nm and 0.67 nm, respectively. (**a**) SHG spectra of monolayer WS_2_, 28L NbOI_2_, and 28L NbOCl_2_. The $\chi^{\left(2\right)}$ of NbOI_2_ and NbOCl_2_ were calculated to be 0.13 and 0.1 times lower than WS_2_, respectively. (**b**) For the THG spectra of monolayer WS_2_, 30L, and NbOI_2_, the $\chi^{\left(3\right)}$ of NbOI_2_ was calculated to be 0.32 times lower than WS_2_.

To quantify the third-order nonlinear susceptibility of NbOI_2_ and TaOI_2_, we measured the THG of NbOI_2_ and TaOI_2_ at different thickness levels using a λ = 1560 nm excitation and benchmarked against monolayer WS_2_, as shown in Figure 3a. The THG signal of 22 nm NbOI_2_ exceeds that of the WS_2_ monolayer by two orders of magnitude under identical conditions. Following established procedures [33], the second- and third-order nonlinear susceptibility can be estimated by the measured averaged power for the same incident polarized excitation and crystal orientation. The third-order effective nonlinear susceptibility can be described as follows [39]:

(2) $$\chi_{eff}^{\left(3\right)}=\frac{\chi_{s}^{\left(3\right)}}{d}=\sqrt{\frac{P_{THG}\left(3\omega \right)c^{4}\varepsilon_{0}^{2}{\left(ft_{fwhm}\pi r^{2}\right)}^{2}{\left(1+n_{2}\right)}^{8}}{64\sqrt{3}S^{2}\omega^{2}P_{pump}^{3}\left(\omega \right)d^{2}}}$$

Here, $P_{THG}\left(3\omega \right)$ and $P_{Pump}\left(\omega \right)$ are the average *TH* and pump power, *c* is the speed of light in vacuum, *f* is the pump repetition rate, *t_fwhm_* is the pulse width, *r* is the focal spot radius, $n_{2}$ is the substrate refractive index, *S* is a shape factor for Gaussian pulses, which describe the temporal intensity distribution of the pulses, *ω* is the fundamental frequency, $\chi_{s}^{\left(3\right)}$ is the third-order sheet nonlinear susceptibility, and *d* is the sample thickness. Similarly, the second-order effective nonlinear susceptibility $\chi_{eff}^{\left(2\right)}$ can be described as:

(3) $$\chi_{eff}^{\left(2\right)}=\frac{\chi_{s}^{\left(2\right)}}{d}=\sqrt{\frac{P_{SHG}\left(2\omega \right)c^{3}\varepsilon_{0}ft_{fwhm}\pi r^{2}{\left(1+n_{2}\right)}^{6}}{16\sqrt{2}S\omega^{2}P_{pump}^{2}\left(\omega \right)d^{2}}}$$

Here, $P_{SHG}\left(2\omega \right)$ is the average *SH* power. From the *SHG* results, we deduced the effective second-order susceptibility of monolayer WS_2_ to be around 500 pm/V at the fundamental wavelength of 800 nm, which is in reasonable agreement with the reported results [34]. The third-order susceptibility at 1560 wavelength is $3.2\times 10^{-19}\;m^{2}/V^{2}$, which is consistent with the value from ref. [40] under similar excitation wavelength. These enable the direct extraction of susceptibilities, based on Equations (2) and (3). Alternatively, unknown materials can be evaluated directly by analyzing the output spectra from the same experiments. 

In a more practical approach, nonlinear optical susceptibility can be quantified by comparing the *THG* spectra acquired under the same experimental conditions. The following relation enables the evaluation of the third-order nonlinear susceptibility of MOI_2_(M = Nb, Ta) based on the obtained value of monolayer *WS*_2_:

(4) $$\frac{\chi_{eff-MOI_{2}}^{\left(3\right)}}{\chi_{eff-WS_{2}}^{\left(3\right)}}=\sqrt{\frac{P_{THG-MOI_{2}}\left(3\omega \right)}{P_{THG-WS_{2}}\left(3\omega \right)}}\frac{t_{WS_{2}}}{t_{MOI_{2}}}$$

This allows the third-order effective nonlinear susceptibility to be determined readily from the measured spectra. Here, as an example, by comparing the SHG intensity of NbOI_2_ with monolayer *WS*_2_ and using the deduced $\chi^{\left(2\right)}$ of *WS*_2_ at 800 nm, we get $\chi_{eff-NbI_{2}}^{\left(2\right)}\sim 65\;\mathrm{pm}/V$ for NbOI_2_, consistent with the recent reports [28], which is one-order magnitude higher than 3D nonlinear optical crystals [41,42,43]. From the acquired THG spectra of the 22 nm NbOI_2_ and monolayer WS_2_ in Figure 3b, Equation (4) permits the direct extraction of the $\chi^{\left(3\right)}$. When compared to monolayer WS_2_, the THG of monolayer WS_2_ is equivalent to the NbOI_2_ with a thickness of 1.78 layers and TaOI_2_ with a thickness of 1.81 layers. The effective susceptibility can exclude the influence of thickness and better describe third-order nonlinearity. Thus, we obtain $\chi_{eff-NbOI_{2}}^{\left(3\right)}=0.75\times 10^{-19}\;m^{2}/V^{2}$ and $\chi_{eff-TaOI_{2}}^{\left(3\right)}=0.73\times 10^{-19}\;m^{2}/V^{2}$. These values are summarized in Table 1, alongside relevant references.

**Figure 4:**
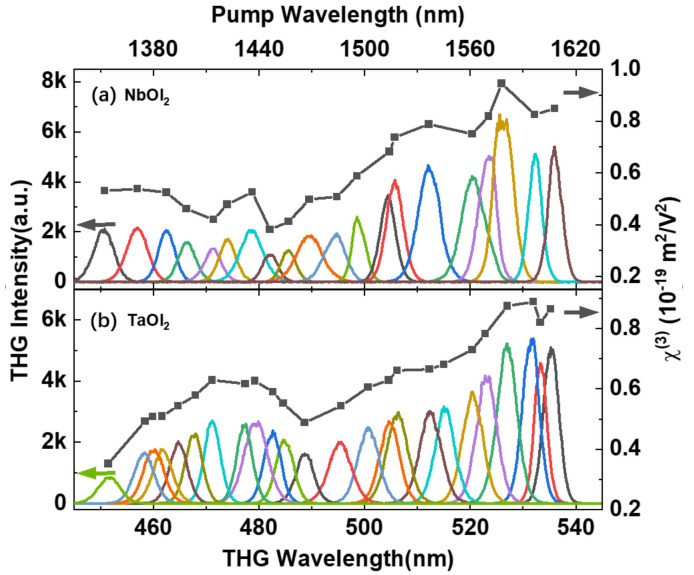
THG emission spectroscopy (left panel, spectra in colorful lines, guided by left arrows) and third-order nonlinear susceptibility $\chi^{\left(3\right)}$ (right panel, black squares, guided by right arrows) of (**a**) 22 nm-NbOI_2_ and (**b**) 20 nm-TaOI_2_ at the fundamental excitation wavelength range of 1350 to 1605 nm. All the measurements were taken with the same average excitation power. Top panel corresponds to the fundamental excitation wavelength.

Optical harmonic generation could be enhanced when the energy is in resonance with the band edge or excitonic states [1,6,47,48]. In graphene the one-, two-, and three-photon processes can participate in the final THG together due to linear dispersion. Thus, the tuning of the Fermi level controls the third-order process over orders of magnitude [49,50,51]. To further examine the wavelength dependence in MOI_2_, we systematically varied the excitation wavelength in the telecommunications range from 1350 to 1605 nm, at a fixed excitation power of 5 mW (Figure 4a,b). For NbOI_2_, the THG intensity increases monotonically with wavelength, reaching its maximum at 1595 nm, approximately six times that under 1445 nm excitation. This trend qualitatively agrees with the increase in SHG intensity from 400 to 525 nm [28]. This implies that the THG enhancement possibly stems from similar excitonic resonance effects. Given the bandgap of 2.24 eV estimated in NbOI_2_ [28], both the one-photon fundamental excitation and the two-photon process lie far below the gap across the studied wavelengths. Thus, the resonant enhancement most plausibly arises from the three-photon resonance with the band edge. In contrast, TaOI_2_ exhibits distinct THG enhancement peaks at around 470 nm and 530 nm, respectively, and approximately 3.2 and 6.4 times the THG intensity at 450 nm. While the details of the band structure of TaOI_2_ are still lacking, further theoretical analysis is crucial to fully understand the wavelength-dependent $\chi^{\left(3\right)}$ properties of this material.

As shown in the right panel of Figure 4a,b, the effective third-order nonlinear susceptibility $\chi_{eff}^{\left(3\right)}$ was extracted at different excitation wavelengths. The $\chi_{eff}^{\left(3\right)}$, ranging from 0.38 to $0.94\times 10^{-19}m^{2}/V^{2},$ is comparable with the reported 2D materials (e.g., hBN, graphene and BP) [44,45,50] and traditional nonlinear media such as silicon and silicon nitride [46,52].

**Figure 5:**
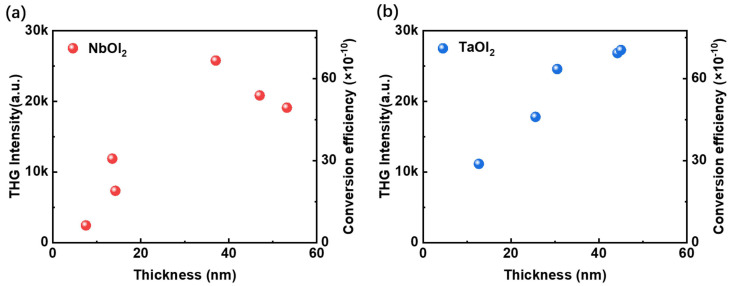
THG responses and conversion efficiency as a function of thickness. (**a**) NbOI_2_ and (**b**) TaOI_2_ with a thickness range of 7.5 to 53.2 nm and 12.7 to 45 nm, respectively.

Figure 5 shows the thickness dependence of THG intensity and conversion efficiency of NbOI_2_ and TaOI_2_ flakes. The signal increases up to ~30 nm, after which it either exhibits saturation or a decrease at larger thicknesses. At the thin thickness limit, the ideal model predicts that the intensity of the harmonic generation scales quadratically below the coherence length and the penetration depth [30]. However, more practical effects, such as interference of the signal from the surface and other depths in the material, absorption of the fundamental light and refractive index at different wavelengths will deviate from the quadratic trend [26,27,28]. Here, we observed THG efficiency increase by orders of magnitude in both materials going from few-layer to multilayer flakes. Notably, the larger overall efficiency can be achieved using transmission geometry due to the larger coherent length [53].

## 4. Conclusions

In conclusion, we have investigated the THG in 2D transition metal oxide dihalides, specifically NbOI_2_ and TaOI_2_. While negligible SHG is detected from the centrosymmetric TaOI_2_, the extracted effective $\chi^{\left(3\right)}$ of these two materials is on the order of $\sim 10^{-19}\;m^{2}/V^{2}$ for both materials under 1580 nm excitation. By tuning the fundamental excitation wavelength across the telecommunications band range, we further obtain the enhancement peak of the third-order susceptibility when in resonance with the band edge, being consistent with the reported band structure qualitatively. The increase in THG with thickness below ~30 nm was revealed due to the unique symmetry and weak interlayer coupling. This provides thickness-based nonlinear efficiency tuning in telecommunications band wavelength. Our results reveal details of the third-order nonlinear process in the 2D NbOI_2_ and TaOI_2_ towards possible applications in integrated all-optical information processing, wavelength conversion, and optical modulation at the on-chip level.

## Figures and Tables

**Table 1:** Effective $\chi^{\left(3\right)}$ of 2D materials.

Material	THG Wavelength (nm)	$\chi^{\left(3\right)}$ $\left(10^{-19}\;m^{2}/V^{2}\right)$	$\eta^{\;*}$ $\left(\times 10^{-10}\right)$	Thickness	Substrate	Reference
NbOI_2_	450–535	0.4–0.9	22.9–114 (0.75)	22 nm	SiO_2_/Si	This work
TaOI_2_	450–535	0.3–0.9	9.5–94.7 (0.75)	20 nm	SiO_2_/Si	This work
WS_2_	520	3.2	1.4 (0.75)	ML	SiO_2_/Si	This work
WS_2_	520	2.4	28 (2.7)	ML	SiO_2_/Si	[40]
hBN	360	0.084	5.3 (3.6)	37 nm	Fused silica	[44]
BP	519	1.4	6 (8.1)	14.5 nm	SiO_2_/Si	[45]
Graphene	520	1	5.4 (1.6)	ML	Glass	[39]
Silicon nitride	355	0.28	0.2 (143)	200 nm	Fused silica	[46]

* Conversion efficiency $\left(\times 10^{-10}\right)$ (excitation peak power, unit: kW).

## Data Availability

The data presented in this study are available on request from the corresponding author.

## Supplementary Materials

The following supporting information can be downloaded at: https://www.mdpi.com/article/10.3390/nano14050412/s1, Figure S1. Experimental setup; Figure S2. XRD of the synthesized NbOI_2_ and TaOI_2_ single crystals.
