# Supplementary material for: Third Harmonic Generation in Thin NbOI2 and TaOI2
Source: Nanomaterials (Basel). 2024 Feb 23;14(5):412. doi: 10.3390/nano14050412 (PMC10934621; doi:10.3390/nano14050412)
Supplement: Supplementary file 1 [file nanomaterials-14-00412-s001.zip › nanomaterials-2875609-supplementary-done.pdf]

nanomaterials

Supplemenatry Materials

Third Harmonic Generation in Thin $NbOI_2$ and $TaOI_2$

Tianhong Tang [1,2], Deng Hu [1,2], Di Lin [1,2], Liu Yang [1,2], Ziling Shen [1,2], Wenchen Yang [1,2], Haiyang Liu [1,2], Hanting Li [1,2], Xiaoyue Fan [1,2], Zhiwei Wang [1,2,*] and Gang Wang [1,2,*]

1 Centre for Quantum Physics, Key Laboratory of Advanced Optoelectronic Quantum Architecture and Measurement (MOE), School of Physics, Beijing Institute of Technology, Beijing 100081, China
2 Beijing Key Lab of Nanophotonics and Ultrafine Optoelectronic Systems, Beijing Institute of Technology, Beijing 100081, China
* Correspondence: zhiweiwang@bit.edu.cn (Z.W.); gw@bit.edu.cn (G.W.)

Citation: Tang, T.; Hu, D.; Lin, D.; Yang, L.; Shen, Z.; Yang, W.; Liu, H.; Li, H.; Fan, X.; Wang, Z.; et al. Third Harmonic Generation in Thin $NbOI_2$ and $TaOI_2$. *Nanomaterials* **2024**, *14*, 412. https://doi.org/10.3390/nano14050412

Academic Editor: Alexander Tselev

Received: 31 January 2024
Revised: 18 February 2024
Accepted: 21 February 2024
Published: 23 February 2024

Copyright: © 2024 by the authors. Licensee MDPI, Basel, Switzerland. This article is an open access article distributed under the terms and conditions of the Creative Commons Attribution (CC BY) license (https://creativecommons.org/licenses/by/4.0/).

Experimental setup

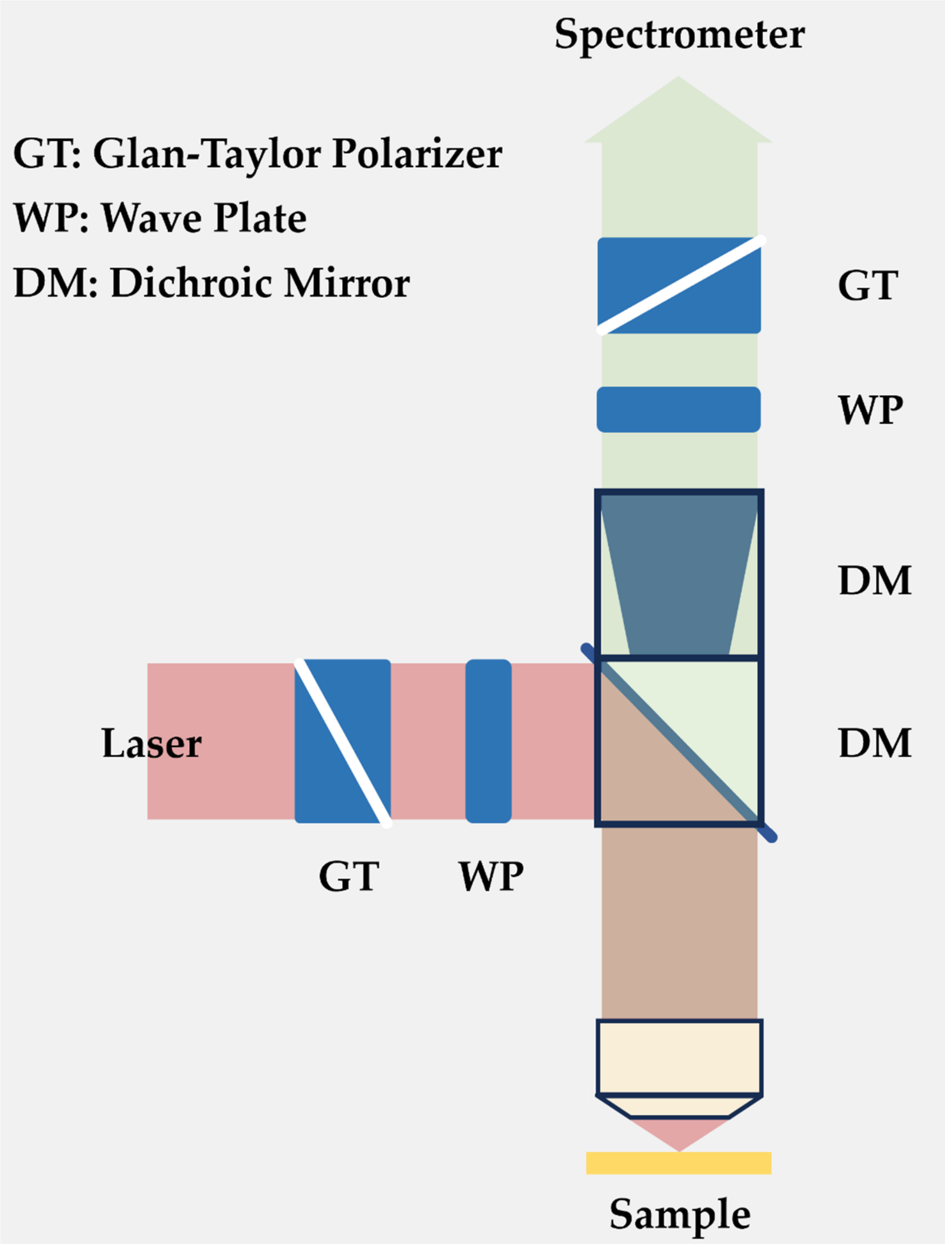

Figure S1. Experimental setup for the THG and SHG measurement.

XRD measurements

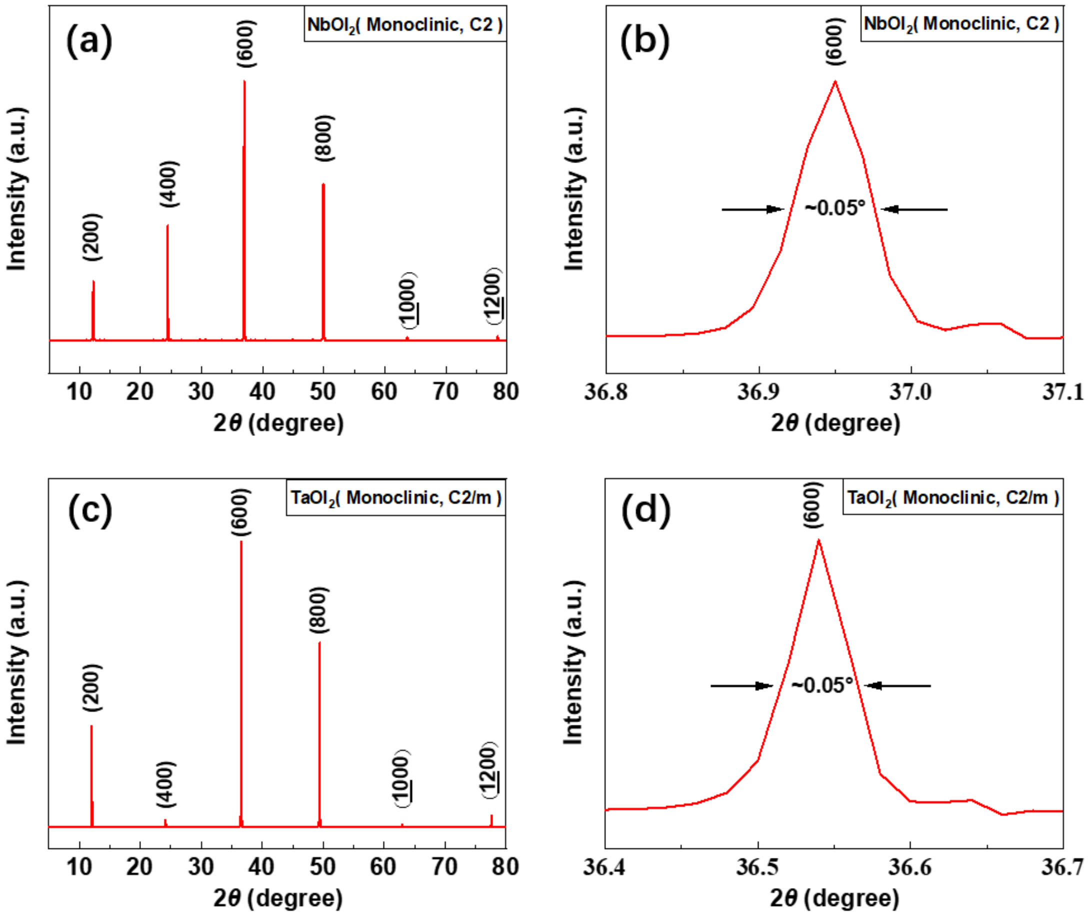

Figure S2. XRD of the synthesized NbOI2 and TaOI2 single crystal. (**a**). XRD pattern of as-grown NbOI2 single crystal. (**b**). Enlarged view of the (600) peak in figure S1a shows, the FWHM is only 0.05°. (**c**). XRD pattern of as-grown TaOI2 single crystal. (**d**). Enlarged view of the (600) peak in figure S2c, the FWHM is only 0.05°.

Disclaimer/Publisher's Note: The statements, opinions and data contained in all publications are solely those of the individual author(s) and contributor(s) and not of MDPI and/or the editor(s). MDPI and/or the editor(s) disclaim responsibility for any injury to people or property resulting from any ideas, methods, instructions or products referred to in the content.
